# Aeromagnetic anomalies reveal the link between magmatism and tectonics during the early formation of the Canary Islands

**DOI:** 10.1038/s41598-017-18813-w

**Published:** 2018-01-08

**Authors:** Isabel Blanco-Montenegro, Fuensanta G. Montesinos, José Arnoso

**Affiliations:** 1 Universidad de Burgos, Departamento de Física, Escuela Politécnica Superior, Avda. de Cantabria s/n, 09006 Burgos, Spain; 2 Facultad de Matemáticas, Universidad Complutense de Madrid, Plaza de Ciencias 3, 28040 Madrid, Spain; 3 Instituto de Geociencias (CSIC, UCM), Facultad de Medicina (Edificio Entrepabellones 7 y 8), Doctor Severo Ochoa 7, 28040 Madrid, Spain; 4 Research Group ‘Geodesia’, Facultad de Matemáticas, Universidad Complutense de Madrid, Plaza de Ciencias 3, Madrid, 28040 Spain

## Abstract

The 3-D inverse modelling of a magnetic anomaly measured over the NW submarine edifice of the volcanic island of Gran Canaria revealed a large, reversely-magnetized, elongated structure following an ENE-WSW direction, which we interpreted as a sill-like magmatic intrusion emplaced during the submarine growth of this volcanic island, with a volume that could represent up to about 20% of the whole island. The elongated shape of this body suggests the existence of a major crustal fracture in the central part of the Canary Archipelago which would have favoured the rapid ascent and emplacement of magmas during a time span from 0.5 to 1.9 My during a reverse polarity chron of the Earth’s magnetic field prior to 16 Ma. The agreement of our results with those of previous gravimetric, seismological and geodynamical studies strongly supports the idea that the genesis of the Canary Islands was conditioned by a strike-slip tectonic framework probably related to Atlas tectonic features in Africa. These results do not contradict the hotspot theory for the origin of the Canary magmatism, but they do introduce the essential role of regional crustal tectonics to explain where and how those magmas both reached the surface and built the volcanic edifices.

## Introduction

**Figure 1:**
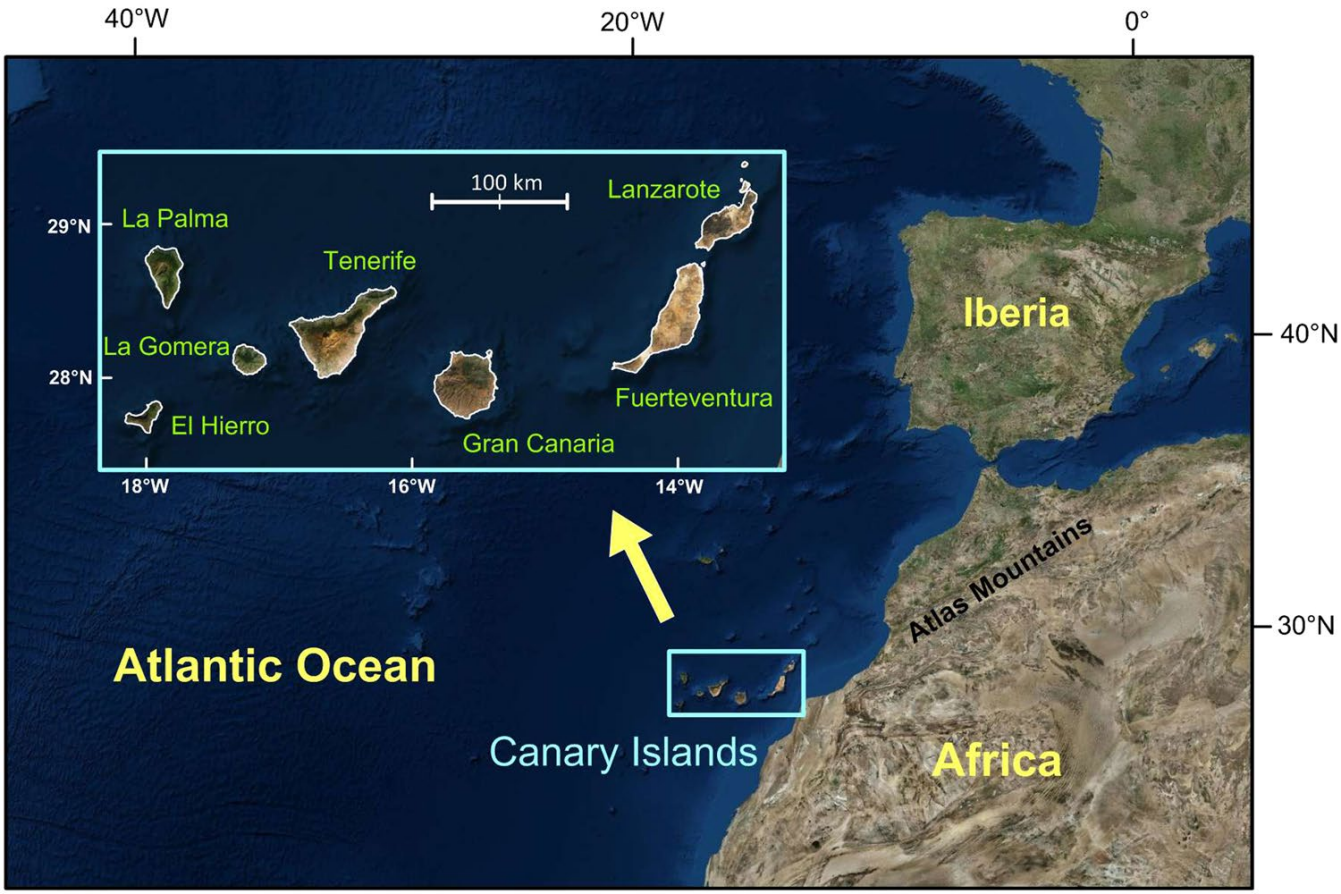
Location of the Canary Archipelago in the Atlantic Ocean near the African coast and details of the Canary region wherein the seven major islands are identified. Gran Canaria is located in the central part of the archipelago between Tenerife and Fuerteventura. The geographical position of the Atlas Mountains in Africa is also shown. Maps generated using Global Mapper v15.1.5 (www.bluemarblegeo.com) and Surfer 11 (www.goldensoftware.com).

The Canary Archipelago is a group of seven islands located in the central North Atlantic (at latitudes from N27°30′ to N29°30′ and longitudes from W18°15′ to W13°15′, see Fig. 1) that constitute a unique case of intraplate volcanism with activity spanning (at least) from the Oligocene until the present time. The early building of these islands occurred within the framework of the opening of the Atlantic and the convergence between the African and European plates. Several genetic theories were proposed during the last 40 years, although each of them failed in some aspect, especially as the available geological and geophysical information has increased. One of those hypotheses claimed a hotspot origin for the Canary volcanism^1–3^, whereas others invoked a propagating fracture from the Atlas Mountains^4^ or mantle decompression melting together with the uplift of tectonic blocks^5^.

The first large-scale seismic tomography studies, which were carried out in the 90′-s^6,7^, revealed the existence of a thermal anomaly in the mantle extending from the eastern Atlantic Ocean to beneath central Europe. More recent global seismic tomography modelling results^8,9^ imaged this thermal anomaly, and showed that the volcanism of the Azores, Canary, and Cape Verde Islands are associated with three adjacent deep plumes that appear as isolated anomalies down to a depth of approximately 1,000 km, where upon they merge and bend eastward with increasing depth and reach the base of the mantle off the coast of Africa beneath the Canary region at approximately 20–25°N.

Since the mantle thermal anomaly origin for the Canary magmatism has been clarified, the key point to be elucidated is to discern how those magmas have reached the surface and whether previous crustal tectonic features have played a significant role in the genesis and early evolution of the Canary Islands.

In an attempt to integrate all of the recent data within an updated approach from a wide perspective, Anguita & Hernán^10^ proposed a unifying hypothesis for the genesis of the Canary Archipelago that points to a strong connection between magmatism and tectonics during its origin. They considered that the islands’ magmatism could be explained through the tapping of a thermal anomaly by fractures inherited from a Mesozoic failed rift arm.

However, this issue is still a matter of intense debate^11,12^. Based on the paradigmatic example of the intraplate volcanism of Hawaii, some authors still claim that a pure hotspot hypothesis is the only explanation for the Canarian magmatism^11,13^. With this paper, we demonstrate using aeromagnetic data that tectonic features present in the crust strongly conditioned the ascent and emplacement of magmas during the early formation of Gran Canaria.

A causal relationship between tectonics and magmatism has been evidenced for Fuerteventura^14–17^, Lanzarote^18^ and Tenerife^19,20^ from studies of volcanic structural elements (e.g. dykes, faults, and vents) and numerical modelling. Indirect evidence of this interplay has been established from geophysical studies and, in particular, from potential field anomalies. These types of data can supply very relevant information about the inner structure of ocean island volcanoes. Specifically, they can help us understand the formation mechanisms of the initial seamounts and submarine volcanoes and establish the constraints under which the early islands’ growth occurred. Information about the submerged portions of oceanic islands is always very scarce or absent, although they represent more than 90% of the islands’ total volumes^21^. The analysis and modelling of magnetic and gravity anomalies have significantly contributed to a better understanding of the structure and evolution of some of the Canary Islands^22–30^. In particular, the sources of the magnetic anomalies were identified as intrusive bodies emplaced during the early phases of the islands’ growth and as the feeding systems of later volcanic stages. On many occasions, these structures display elongated shapes that suggest tectonic controls on their emplacement.

**Figure 2:**
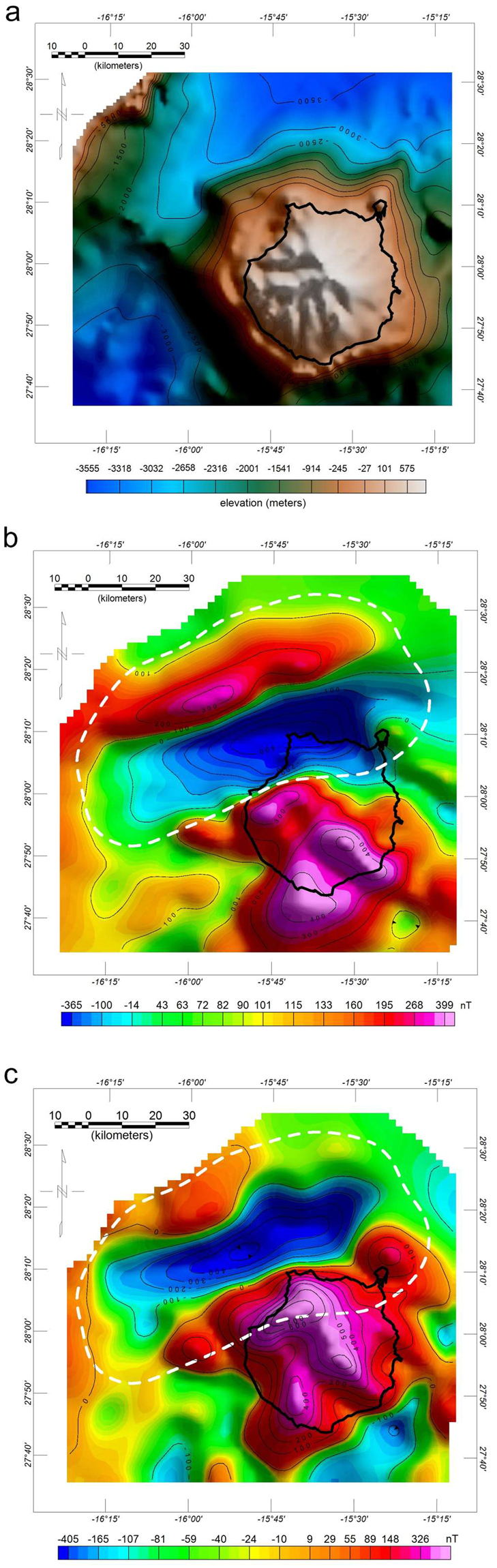
(**a**) Bathymetry and topography of Gran Canaria based on ETOPO1 data. (**b**) Aeromagnetic anomaly map of Gran Canaria at a height of 3,200 m asl. The magnetic anomaly studied here is the reverse dipole displayed over the NW submarine portion of the island. The dashed white line shows the boundary used to extract the data from the aeromagnetic grid for modelling. (**c**) Reduced-to-the-pole aeromagnetic anomaly map of Gran Canaria assuming that the magnetization is parallel to the geocentric axial dipole magnetic field (D = 0°, I = 48°) whereas the Earth’s magnetic field direction (D = −8°, I = 38°) is given by the International Geomagnetic Reference Field^36^. The reverse dipole is transformed into a low in the reduced-to-the-pole magnetic anomaly map, revealing a source body characterized by a negative magnetization contrast. Maps generated with Oasis montaj version 8.1 (www.geosoft.com).

In this work, we conducted the detailed modelling and analysis of an outstanding magnetic anomaly over the marine area to the NW of Gran Canaria (Fig. 2), which is an island located in the central part of the Canary Archipelago between Tenerife and Fuerteventura (Fig. 1). This anomaly was revealed both by aeromagnetic surveying^31^ and marine magnetic data^32^. Due to the vector nature of magnetization (i.e. the physical property responsible for the rocks’ magnetic fields) and, more specifically, its inclination (the angle measured from the horizontal plane), magnetic anomalies show a dipolar form composed of a high and a low. The magnetic anomaly studied here is a reverse dipole (with a low to the south and a high to the north, which is the opposite of a normal dipole in the Earth’s northern hemisphere), implying the presence of a source body characterized by a negative magnetization contrast with respect to the surrounding rocks, suggesting that it was magnetized during a period of reverse polarity of the Earth’s magnetic field. Another remarkable property of this anomaly is its linear characteristics, with straight, parallel contours defining the magnetic gradient between the high and the low. This anomaly pattern reflects the source geometry, revealing an elongated shape (narrow and long) for the causative body.

The magnetic data set used in this work is part of the aeromagnetic survey of the Canary Archipelago performed in 1993 by the National Geographic Institute of Spain^31^. In a previous paper, the magnetic anomaly map of Gran Canaria was analysed through 2.75-D forward modelling and the main magnetic anomalies displayed over the subaerial volcanic edifice of Gran Canaria were associated with intrusive bodies and with the magmatic feeding system of the island^24^.

Here, our objective was to study in detail the reverse dipole anomaly over the marine part of the volcanic edifice of Gran Canaria because of the relevant tectonic implications of such a structure. The development of an advanced 3-D inverse method based on a genetic algorithm^33^ enabled a significant increase in the quality of the modelling of this magnetic source, which we interpret as a magmatic intrusion emplaced during the building of the submarine edifice of Gran Canaria. This anomaly was used as an example in a recently published methodological paper in which our 3-D inverse method was presented^33^. In this study, we refined those earlier modelling efforts and provided an in-depth discussion of the implications derived from our fresh results. We produced a 3-D reconstruction of the shape and location of this large magmatic intrusion, which was interpreted within the geodynamical, geophysical and structural framework of the Canary region; that reconstruction allowed us to establish an important understanding of the mechanisms that caused the initial building of the Canary Islands.

## Data and Methods

The data used in this work were acquired in 1993 by the National Geographic Institute of Spain (IGN) during an aeromagnetic survey of the Canary Archipelago. The details of the survey and the data processing scheme can be found in Socías & Mézcua^31^. The target anomaly covers an area of approximately 80 × 40 km^2^ and ranges from −600 nT to 250 nT (Fig. 2). For the modelling, we assumed that its source can be approximated by a constant magnetization body (both in the intensity and direction). This is a reasonable hypothesis based on the fact that local, small-scale heterogeneities are expected to be averaged and ‘filtered’ when the associated magnetic anomaly is sampled at intervals on the order of 1 km and measured at a height of several kilometres above the source, such as in the data set studied here.

The magnetization of a rock (**J**) is a vector quantity resulting from the sum of two terms: the remanent magnetization (**J**_**R**_), which is parallel to the Earth’s magnetic field at the time of the magnetization acquisition, and the induced magnetization (**J**_**I**_), which is parallel to the present-day Earth’s magnetic field. Volcanic rocks usually acquire their remanent magnetization (called thermoremanence) when they cool below the Curie temperatures of the magnetic minerals.

Taking into account the high Koenigsberger ratios (J_R_/J_I_) typical of volcanic rocks^34^, we assumed that the main component of the total magnetization vector is the remanent magnetization **J**_**R**_ and that the source magnetization **J** is nearly parallel to the remanent magnetization **J**_**R**_.

When the acquisition of J_R_ spans more than 10^5^ years, the secular variation of the Earth’s magnetic field is averaged and the paleomagnetic geocentric axial dipole hypothesis applies. A volcanic structure such as the one studied here certainly required a time span longer than 10^5^ years for its emplacement and cooling. This means that the remanent magnetization **J**_**R**_ can be assumed to be parallel to the geocentric axial dipole magnetic field in Gran Canaria, with a declination D = 0° and an inclination I = 48° (latitude 28°N). Although we made a first estimation of the magnetization direction^33^ using the method of Nicolosi *et al*.^35^, we have now considered that the geocentric axial dipole direction is the most reliable hypothesis for the definition of the magnetization vector.

Therefore, for the inversion, we assumed that the source magnetization was defined by the direction given by a declination D = 0° and an inclination I = 48°, whereas the present-day Earth’s magnetic field has a direction given by a declination D = −8° and an inclination I = 38°, which was obtained from the International Geomagnetic Reference Field^36^ in Gran Canaria for the epoch 1993.8 (i.e., when the aeromagnetic survey was carried out).

Studies of rock magnetic properties in Gran Canaria were conducted for shield basaltic lava flows and for sediments from the volcanic apron^37–41^ but not for intrusive bodies. For instance, Leonhardt *et al*.^39^ studied 557 samples from 87 lava flows from the shield basaltic stage (mid-Miocene age) and obtained natural remanent magnetization (NRM) intensities in the range from 0.2 to 9.5 A/m, with an average value of 1.9 A/m ± 1.4 A/m. Since it was not possible to constrain the magnetization intensity (the length of the total magnetization vector *J*), based on paleomagnetic determinations for intrusive rocks, we worked with values of *J* comprised between 1 and 10 A/m, which are in range typical for volcanic rocks^34^ and agree with the results mentioned before^39^. When we refer to the magnetization intensity, we actually represent the magnetization contrast, since the inversion algorithm searches for a magnetic body surrounded by a volume characterized by a different magnetization.

In Fig. 2c we show the reduced-to-the-pole magnetic anomaly map of Gran Canaria obtained assuming a magnetization direction parallel to the geocentric axial dipole magnetic field (D = 0°, I = 48°). Magnetic anomalies at the magnetic pole, where the field is vertical, lose their dipolar shape (associated high and low) and are transformed into a high (when the magnetization contrast of the source body is positive) or a low (when the magnetization contrast of the source body is negative). Our target magnetic anomaly is transformed into a low in the reduced-to-the-pole magnetic anomaly map, revealing a source body characterized by a negative magnetization contrast.

The negative sign of the magnetization contrast can be explained by: (1) a non-magnetic body surrounded by normally magnetized rocks; (2) a normally-magnetized body with a magnetization weaker than that of the surrounding rocks; or (3) a reversely-magnetized body surrounded by normally magnetized rocks. From these three possibilities, the geological context supports the hypothesis of a reversely-magnetized body, as we will explain in the Discussion section. This interpretation was also proposed by other authors^32^.

For the modelling, we used a magnetic anomaly grid with a cell size of 2 km defined at a height of 3,200 m, from which we extracted the data inside the boundary shown in Fig. 2b. A delicate issue in the inverse modelling process is the possible interference of the target anomaly with the magnetic fields due to nearby sources. Since the isolation of a magnetic anomaly from a magnetic map through filtering involves the removal of part of the magnetic signal, we chose to tackle this problem by defining a volume for the inversion which was large enough to ensure that the body responsible for the target anomaly was completely contained inside it. With this approach, the magnetic fields due to sources different from the target body are not modelled and remain in the residual magnetic field (i.e., the difference between the measured magnetic anomaly and the model magnetic anomaly).

Therefore, a mesh consisting of 16,349 rectangular prisms (covering the same area as the magnetic anomaly grid) distributed throughout 15 layers from the topographic/bathymetric surface (defined using ETOPO1 data) to a depth of 18,500 m was built for the inversion. The prism size was chosen by considering the 2-km resolution of the magnetic anomaly grid. Each prism has a dimension of 2,000 m × 2,000 m × 1,000 m in the N-S, E-W and vertical directions, respectively, for the layers with depths between 500 m above the sea level (asl) and 10,500 m below the sea level (bsl), while each prism has a dimension of 2,000 m × 2,000 m × 2,000 m for the deepest layers (from 10,500 m bsl to 18,500 m bsl).

The modelling of the source of this magnetic anomaly was accomplished by means of the inverse approach developed by Montesinos *et al*.^33^. The method is based on a genetic algorithm that estimates the geometry and location of a homogeneously magnetized source from a 3-D prismatic partition of the subsoil volume while assuming a constant magnetization within each cell. The input information includes the parameters that define the magnetization vector of the source. The algorithm seeks an optimum solution from an initial population of models (i.e., possible solutions) through an evolutionary process.

Each distribution of magnetization in each stage of the evolution process is a possible model and is therefore a potential solution (good or bad) of the inverse problem. A set of these possible models constitutes a population of individuals that take part in the evolutionary process. The algorithm starts with a population of *np* “empty models” where all of the prisms have a null magnetization. Then, the genetic algorithm performs successive modifications of this population in an iterative process using three operators (selection, mutation and crossover), which look for the minimization of an error function. This error function comprises two terms: (1) the discrepancies between the observed data (measured magnetic anomalies) and the calculated data (magnetic anomalies created by the model) obtained using the forward problem formulation; and (2) a quadratic expression of the model parameters (i.e., the Tikhonov regularization technique^42^).

In the iterative procedure, the individuals of the population are evaluated using the error function, and they can be chosen (i.e. using the selection operator) to continue the evolution process through the actions of two genetic operators (mutation and crossover), which produce new *ℓ* individuals. Then, the selection operator chooses the best *np* individuals from these *np* (old) + *ℓ* (new) models, and the iterative procedure is repeated until a predetermined value of the error function (chosen according to the precision of the data) is reached. Once the error function reaches the minimum, a smoothing operator is employed in each model that assigns an average value of the obtained magnetization to each prism while taking into account the adjacent prisms. This operator produces models with a smooth geometry, and it is equivalent to minimizing the whole source volume. Then, these smoothed, new individuals are evaluated again, and if the best model fits the observed data successfully according to the fixed limit, or if it does not improve upon the previous model after several stages, the inversion ends. Otherwise, the evolution process occurs again. When the process finishes, the best 3-D model of the magnetic sources (i.e., that minimizes the error function) is considered the solution of the magnetic inverse problem. A thorough description of the inversion algorithm can be found in Montesinos *et al*.^33^.

In this methodology, the input information is made up of three parameters that remain constant during the whole process: the direction of the present-day magnetic field of the Earth, the direction of the source magnetization, and the intensity of the source magnetization. The choice of these parameters has been already explained in the preceding paragraphs.

## Results

The models obtained through the inversion of the magnetic data for magnetizations within the range of 2 to 10 A/m are supplied as “Supplementary Material” to be examined by the reader (Figs S1 to S8). The properties of these models are summarized in Table 1 (the lowest value of 1 A/m was discarded because of the poor fit obtained). It can be noticed that the fit quality, which was quantified using the root mean square (rms) error of the residual field (i.e., the difference between the measured anomaly and the model anomaly), decreases from the extreme magnetizations to the central values, reaching its minimum for 3–4 A/m.

**Table 1:** Compilation of the geometric parameters (i.e. bottom depth and volume) characterizing the magnetic inversion models obtained for the magnetizations from 2 to 10 A/m. The fit quality in each case, quantified through the root mean square (rms) error of the residual anomaly (the difference between the measured anomaly and the model anomaly) is also indicated.

Magnetization contrast of the magnetic model	Model bottom depth	Source volume	Residual field (rms error)
2 A/m	Not detected	More than 14600 km^3^	143 nT
3 A/m	12500 m	8080 km^3^	96 nT
4 A/m	9500 m	5412 km^3^	98 nT
5 A/m	8500 m	3808 km^3^	101 nT
6 A/m	6500 m	2820 km^3^	105 nT
7 A/m	5500 m	2088 km^3^	109 nT
8 A/m	5500 m	1628 km^3^	111 nT
9 A/m	5500 m	1084 km^3^	122 nT
10 A/m	4500 m	668 km^3^	138 nT

From a geometrical point of view, it is obvious that the effect of changing the magnetization intensity is to modify the source volume and the depth to the source bottom, i.e., an increase in the magnetization reduces the source volume and raises the bottom surface. For instance, the source bottom is not defined for a value of 2 A/m since the lower layer of the inversion volume still contains non-null magnetization cells. This reveals that an intensity of 2 A/m is too low and that the actual magnetization contrast is surely larger. On the other hand, models for magnetizations higher than 7 A/m reveal non-continuous bodies, demonstrating that those values are too high to represent a bulk magnetization under the assumption of a constant magnetization for the source. This agrees with previous magnetic studies in the Canary Islands, where the intrusive bodies were modelled with magnetizations always lower than 6 A/m^24,25,28,29^.

Taking into account these results, it is reasonable to restrict the interval of possible magnetizations to the range between 3 and 7 A/m. All these models can be considered a possible representation of the causative body. Our interpretation is based on the source properties that shared among all of the models and do not depend strictly on the magnetization intensity assumed. All models reveal an elongated shape for the body responsible for the studied magnetic anomaly, which follows an ENE-WSW direction. The main uncertainty is the depth to the bottom of the source; the source depth does depend upon the magnetization intensity and deepens as the magnetization decreases.

**Figure 3:**
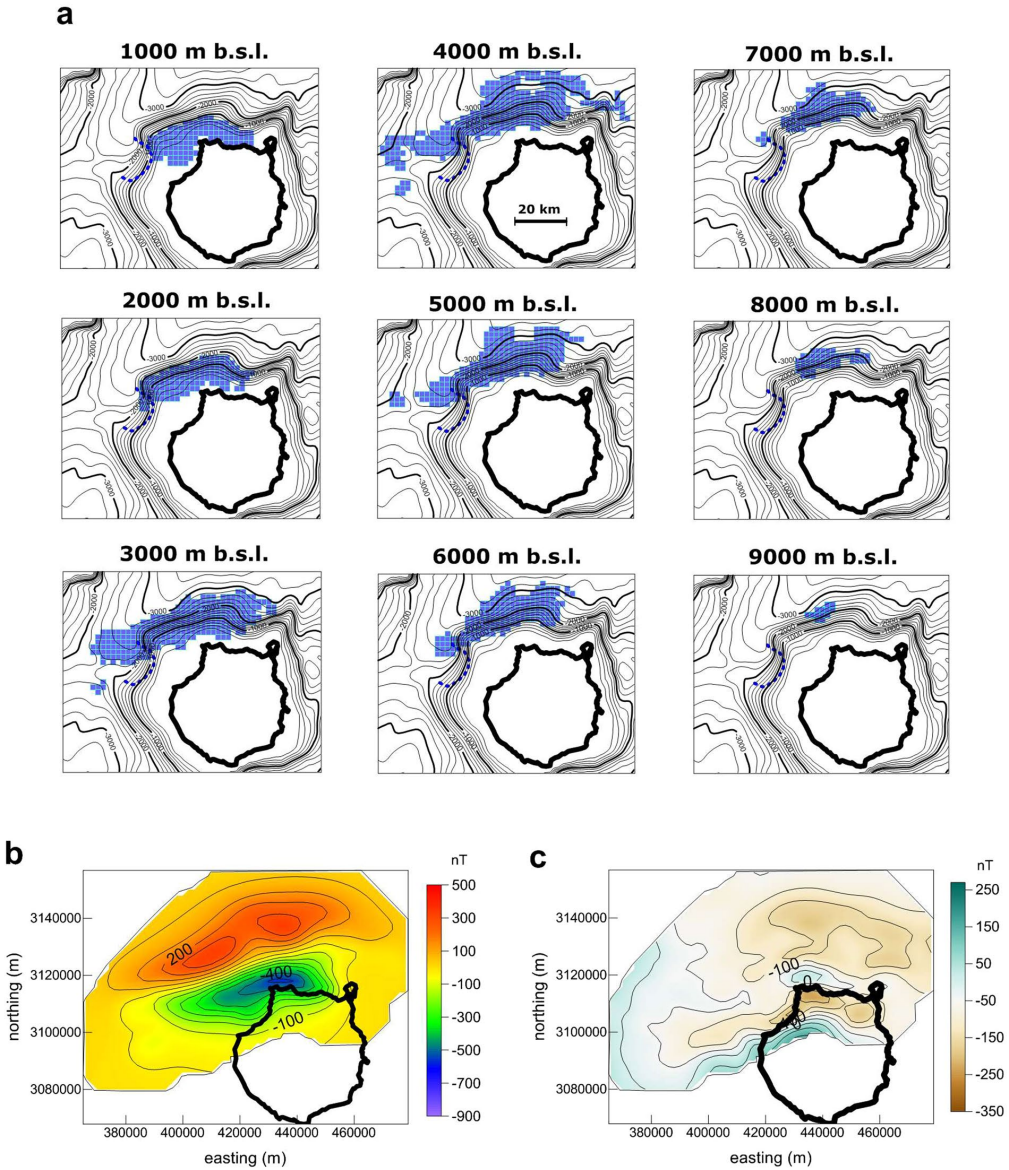
(**a**) Horizontal sections of the magnetic source obtained through the inversion of the aeromagnetic data for a magnetization of J = 4 A/m. The thick black line shows the coastline of the island. The dashed blue line marks the position of the submarine landslide scar identified by Krastel & Schmincke^47^. (**b**) Magnetic anomaly from the inversion model shown in a. (**c**) Residual magnetic anomaly obtained as the difference between the observed magnetic anomaly shown in Fig. 2 and the magnetic anomaly of the inversion model shown in panel b. Coordinates correspond to the UTM projection (zone 28 N). Produced with Surfer 11 (www.goldensoftware.com).

Figure 3 shows the model obtained with a magnetization of 4 A/m, which corresponds to the central part of the estimated magnetization interval and shows geometric properties (horizontal extension and depth range) that are intermediate between the extreme models (compare it with Supplementary Figs S1 to S8). This model reveals an elongated body with a volume of nearly 5.4 × 10^3^ km^3^ that extends from the bathymetric surface to a depth of 9,500 m bsl and measures approximately 80 km x 20 km at a depth of 4,000 m bsl. Horizontally, the source is located over the slope of the NW submarine edifice following an ENE-WSW direction. The same location and orientation can be noted in all models (Supplementary Figs S1 to S8).

## Discussion

### Volcanological interpretation

Two main stages can be identified during the building of a volcanic island^21,43^: an initial stage of submarine growth during which a seamount is formed and a subsequent subaerial stage, which begins when the submarine volcano reaches the water-air discontinuity. Most of the subaerial growth occurs through fissure basaltic eruptions that build rift zones and generate shield volcanoes that comprise more than 90% of the subaerial edifice. In some islands, this initial basaltic activity is followed by other stages (post-shield activity) during which evolved volcanic materials are erupted and eventually form calderas. Volcanic activity is not continuous, and important hiatuses can be identified within the reconstructions of the volcanic histories of ocean island volcanoes, which must be understood as the results of the competition between constructive (both intrusive and extrusive) and destructive processes (e.g., giant collapses and erosion).

The submarine part of a volcanic island constitutes the most important portion of the edifice since it can represent more than 90% of the whole island volume; in most cases, however, it is practically unknown. The total volume of Gran Canaria was estimated in the range of 35–40 × 10^3^ km^3^ ^44^, of which only 824 km^3^ correspond to the subaerial portion^45^, representing 2.0–2.3% of the island.

A substantial part of our knowledge about the inner structure of seamounts is based on the studies carried out on the island of La Palma, which is located in the western part of the Canary Archipelago. There, the outcrop of an uplifted seamount provided an extraordinary opportunity to study this early volcanic stage. It was observed that intrusive and extrusive activity contributed in roughly equal portions to the submarine volcanic construction. In particular, it was observed that the basal extrusive portion of the La Palma seamount (composed of 76% pillow lavas, 5% sheet flows, and 19% clastic rocks) was intruded by a massive sheeted sill swarm, which represents roughly 50% of the seamount series, and feeder dikes^43,46^.

In Gran Canaria, our models revealed the presence of an elongated and narrow body inside the NW portion of the submarine edifice of the island. The body extends vertically from the bathymetric surface to a depth probably in the range between 6 km to 13 km bsl (see Table 1). This structure can be interpreted as a large sill-like magmatic intrusion emplaced during the submarine growth stage. The main sources of the aeromagnetic anomalies observed in other islands of the Canary Archipelago were also interpreted as intrusive complexes ascribed to the early formation of the volcanic edifices^25,28,29^ since intrusive rocks are usually characterized by high magnetization contrasts compared with the surrounding rocks^34^.

The inverse modelling results suggest that the volume of this body probably ranges between 2.1 × 10^3^ km^3^ and 8.1 × 10^3^ km^3^, with a central value of 5.1 × 10^3^ km^3^. This represents a proportion from about 6% to 21% of the total volume of the volcanic edifice (35–40 × 10^3^ km^3^), suggesting that the emplacement of this magmatic body could have been a very relevant event in the volcanic history of Gran Canaria.

When simultaneously analysing the morphology of the NW submarine flank of Gran Canaria (Fig. 2a) and the shape and orientation of the magnetic source (Fig. 3), it can be concluded that both of these features show a linear geometry with an ENE-WSW orientation (note the coherence between the isobath orientations and the trend of the magnetic anomaly). This implies that the magnetic source also has a shallow portion defining the shape of the submarine topography in this area, suggesting that the magnetic body consists of intrusive rocks but also contains a portion of massive submarine pillow lavas. We must stress, however, that even for the highest magnetizations the inverse modelling extends the source below the seafloor (see Supplementary Figs S4 and S5).

Regarding the explanation for the negative magnetization contrast of the magnetic source, we conclude that it is the result of the acquisition of a reverse remanent magnetization during the cooling of the magmatic body. In the shallow portion of the models, the negative magnetization contrast could be explained as the difference between a reverse magnetization (submarine edifice) and a null magnetization (surrounding seawater). In the deep part, the negative magnetization contrast would come from the contact between a reverse magnetization and the weak, normal magnetization of the Jurassic crust where the Canary Islands were built.

It is interesting to note that the linear morphology of the NW part of the submarine flank is interrupted to the SW by a rugged depression delimited by a rounded scarp (Fig. 3). Taking into account that the deep part of the magnetic body extends to the west beyond this depression, we can deduce a) that the extension of this part of the submarine edifice was initially larger and b) that a portion of it was removed by a giant collapse that occurred after the emplacement of the magmatic intrusion. This scarp was mentioned by Krastel & Schmincke^47^, who deduced that the associated collapse occurred at the end of the shield volcano-building phase.

The SE part of the submarine edifice of Gran Canaria also displays a linear shape with a very steep gradient and an ENE-WSW trend, although no associated magnetic anomaly can be identified in this area. This is additional proof that the magnetic anomaly studied here cannot be explained only as the effect of a reversely magnetized submarine topography and that the presence of a magnetized body extending to a depth of several kilometres beneath the seafloor is required to reproduce the measured magnetic anomaly.

Radiometric data showed that the subaerial shield basaltic stage in Gran Canaria was confined to a very short time interval^48–50^. The emission rate for this period was estimated to be nearly 4,200 km^3^/My (see Fig. 2 in Hoernle *et al*.^48^). This rapid growth was confirmed by data acquired in the framework of the *Ocean Drilling Program* from the volcanic apron of the island, wherein a thick layer composed of hyaloclastites and debris flows was found at the base of the drill holes, suggesting that the eruptive rates during the initial and major growth phases were very high^49^.

In an attempt to estimate the time span required for the emplacement of the magmatic intrusion, and taking into account the absence of data regarding the submarine construction, we assumed that the eruptive rate of this initial phase of growth was approximately the same as the eruptive rate during the building of the shield basaltic volcano. Considering the emission rate of 4,200 km^3^/My mentioned before, the time span for the emplacement of 2.1 × 10^3^ km^3^ (source volume for J = 7 A/m) to 8.1 × 10^3^ km^3^ (source volume for J = 3 A/m) of magma would be comprised between 0.5 My and 1.9 My, respectively, with an average value of 1.2 My.

The timing of the submarine stage of growth is unknown, as the only constraints are the ages of the samples belonging to the oldest subaerial outcrops, which are 15–16 Ma^50,51^. Therefore, the magmatic event responsible for the aforementioned intrusion should have taken place prior to that timeline during a time period when the polarity of the Earth’s magnetic field was predominantly reversed. This can be deduced from the associated magnetic anomaly, which is a reverse dipole with a low to the south and a high to the north.

**Figure 4:**
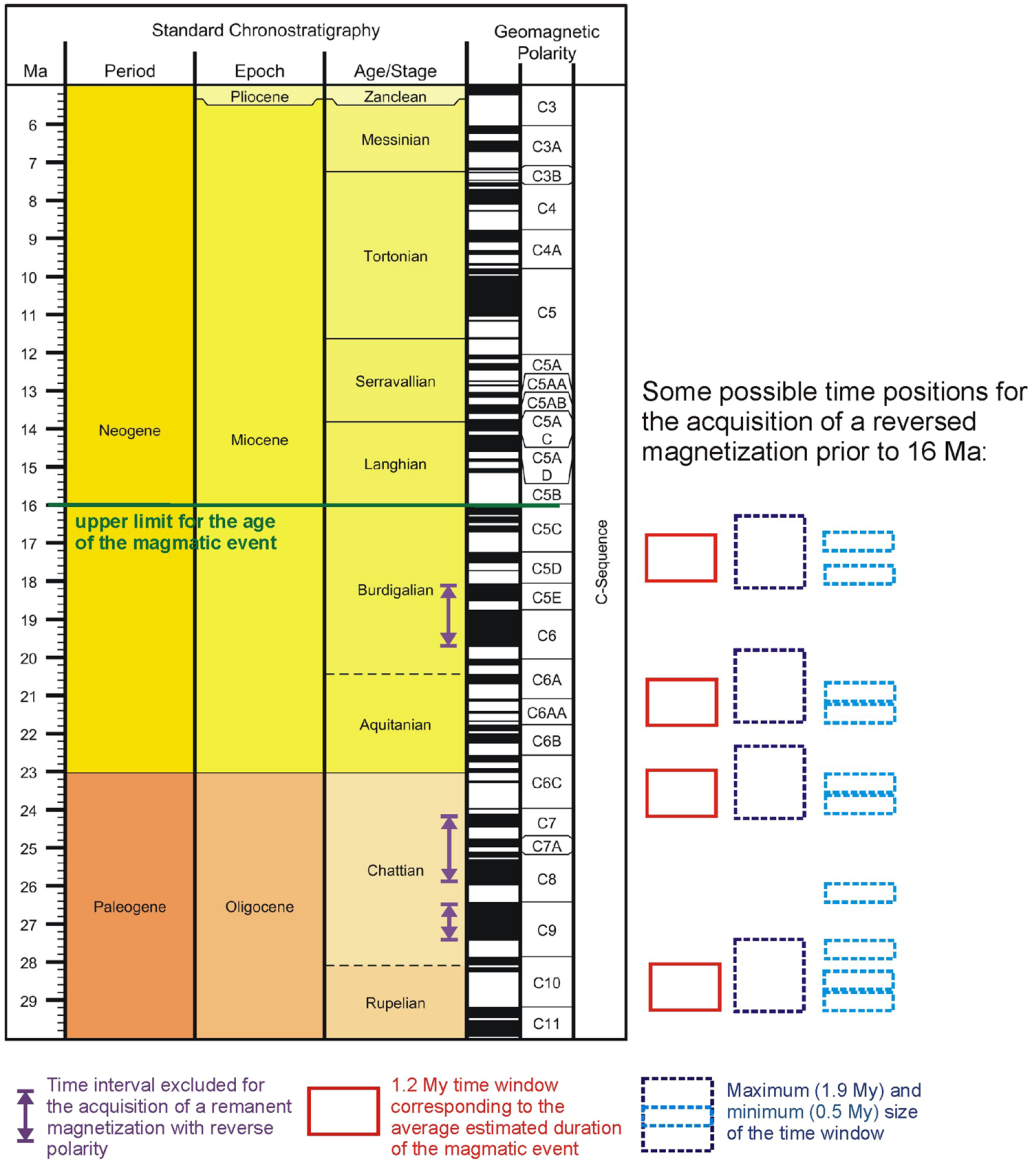
Geomagnetic polarity time scale for the last 30 My based on the Global Time Scale 2012^52^. The green line marks the upper limit for the age of the magmatic event responsible for the emplacement of the magnetic source that is based on the geochronological knowledge of Gran Canaria, which places the beginning of the subaerial shield basaltic stage between 15–16 Ma^50,51^. The rectangles at the bottom of the figure show the sizes of the time windows corresponding to the maximum (1.9 My, in blue), minimum (0.5 My, in cyan) and average (1.2 My, in red) estimated durations of the magmatic event. Several positions of these time windows across the scale are compatible with the acquisition of a reverse magnetization prior to 16 Ma. Three time intervals are excluded for the acquisition of a remanent magnetization with reverse polarity: from 18.0 to 19.7 Ma (chrons C5E and C6), from 24.0 to 26.0 Ma (chrons C7 and C8) and from 26.4 to 27.4 Ma (chron C9), because during those periods the geomagnetic polarity was mainly normal. Produced using TimeScale Creator software version 6.8 (https://engineering.purdue.edu/Stratigraphy/tscreator/index/index.php).

The geomagnetic polarity time scale^52^ shows that in the Upper Oligocene and Miocene, several time periods with durations from 0.5 My to 1.9 My are compatible with a predominant reverse polarity of the geomagnetic field (Fig. 4). Without additional constraints, we cannot establish what position in the time scale is the most likely for the magnetization acquisition. However, it is easy to deduce that the magmatic event did not occur between 18.0 and 19.7 Ma (chrons C5E and C6), between 24.0 and 26.0 Ma (chrons C7 and C8) or 26.4 and 27.4 Ma (chron C9) because, during those time intervals, the polarity was predominantly normal.

### Tectonic implications

As we mentioned before, our models revealed the presence of an elongated and narrow structure that can be interpreted as a sill-like magmatic intrusion emplaced within the submarine edifice of Gran Canaria. This shape is a common property among each of the models and is independent of the assumed magnetization contrast (changing the magnetization contrast mainly affects the source volume) and the magnetization direction (changing the magnetization direction slightly shifts the horizontal position of the source but does not alter its shape). This means that the ambiguities within the modelling procedure have no effect on the volcanological or tectonic interpretation of this structure. Such a geometry clearly points to a structural control on the magma ascension and emplacement and suggests that the growth of Gran Canaria during its submarine phase was strongly conditioned by the presence of a major fault, which favoured the rise of magmas to the surface along a nearly ENE-WSW direction.

The first line of evidence about the existence of a NE-SW trending fault between Tenerife and Gran Canaria was obtained during the seismic study of Bosshard & MacFarlane^53^. These authors also analysed marine gravity profiles and observed that *“the region north of Gran Canaria shows an outstanding feature in the Bouguer anomaly map. Here, the anomaly changes by 138 mgal within 23 km with a very steep gravity gradient in places. This is strong evidence for a fault running perpendicularly to the gradient, i.e. NE to SW”*. The extraordinary agreement between the gravimetric study of Bosshard & MacFarlane and our aeromagnetic models is proof of the presence of such an important tectonic feature in this area and its influence on the island’s early evolution. New gravity data acquired by our research group also confirm this interpretation.

Seismic activity in the Canary region has also been associated with this fault. An important earthquake (with a magnitude of 5.2) occurred in May 1989 in the marine area between Tenerife and Gran Canaria. Mézcua *et al*.^54^ studied the focal mechanism of this event and observed that the principal stress axis was almost horizontal with a NNW-SSE orientation. They interpreted the seismotectonic origin for this earthquake as a sinistral strike-slip fault located between Tenerife and Gran Canaria with a reverse component of motion resulting in the underthrusting of the western block (Tenerife) and a southwest relative displacement of Tenerife with respect to Gran Canaria and Africa. Other authors, however, proposed a volcanic origin for this earthquake^55^.

In addition, the submarine fault between Tenerife and Gran Canaria has been identified as the possible seismotectonic source of a paleoearthquake that occurred 10,000 years ago in the southern part of Tenerife^56^.

**Figure 5:**
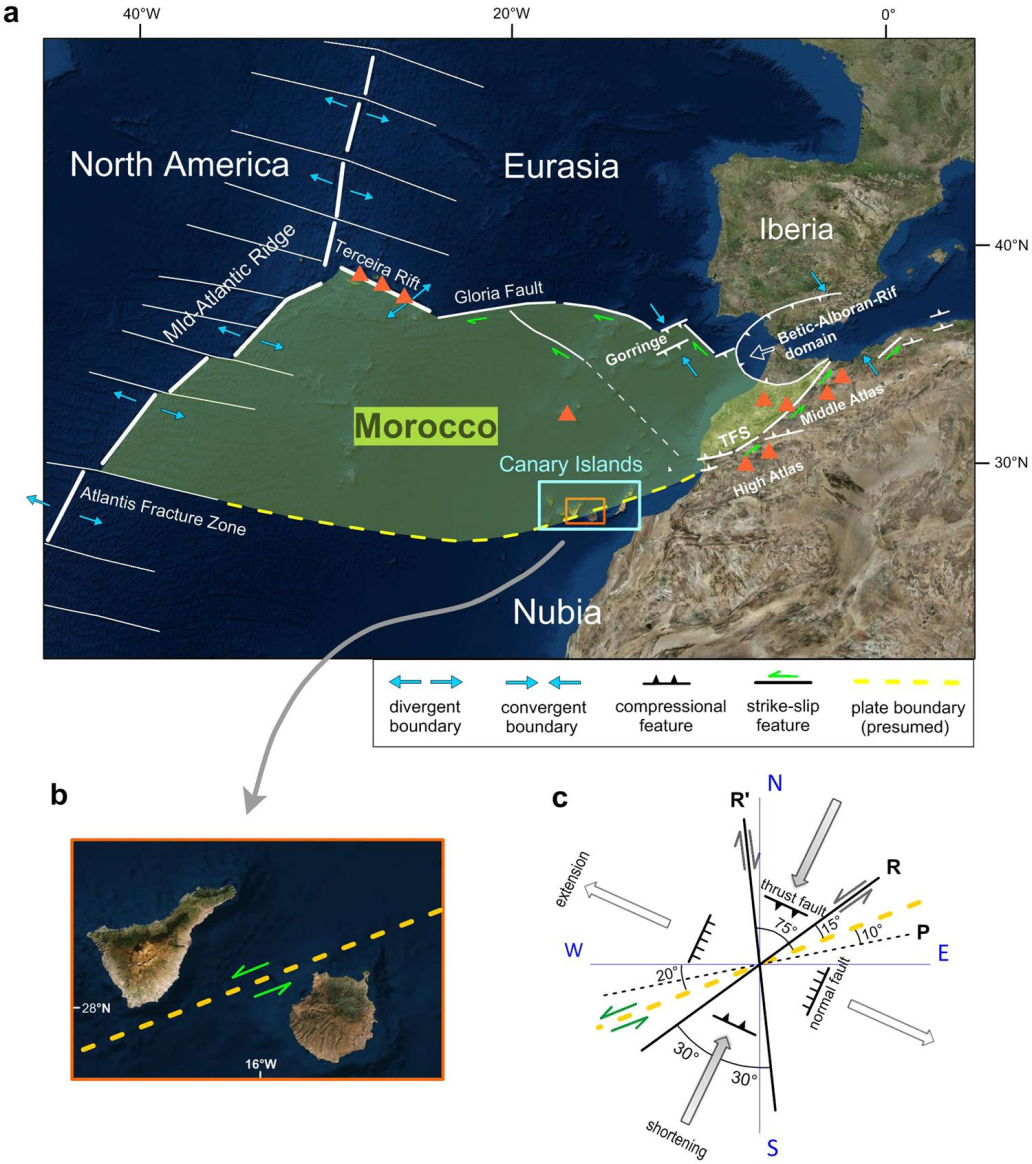
(**a**) Simplified tectonic sketch of the Central Atlantic showing the geometry of the Morocco microplate proposed by Mantovani *et al*.^57^ and indicating the tectonic regimes identified along the plate boundaries. The yellow dashed line marks the presumed southern plate boundary of the Morocco microplate, which crosses the Canary Archipelago between the central islands of Tenerife and Gran Canaria. TFS: Trans-Alboran Fault System. Orange triangles show the locations of Pliocene-Quaternary alkaline-basaltic volcanics in the region outside the Canary Archipelago (the Azores and Madeira Archipelagos in the Atlantic Ocean and the Atlas Mountains in the African continent). Details for the Iberia microplate and the Betic-Alboran-Rif domain are omitted. (redrawn from Mantovani *et al*.^57^); (**b**) Details of the central part of the Canary Archipelago showing the location of the strike-slip fracture segment between the islands of Tenerife and Gran Canaria. Note the extraordinary agreement between the trend of this tectonic feature and the orientation of the magnetic source obtained through our inverse modelling (Fig. 3). (**c**) Fault system developed in a sinistral strike-slip zone such as the Trans-Alboran fault system, that extends from the African continent to the Canary region, with a strike of nearly 70° (thick, yellow dashed line) (based on the scheme of Davis & Reynolds^58^ shown in their Fig. 6.148). Riedel shears (R) are sinistral strike-slip faults that form at an angle of 15° to the main line of faulting (a strike of 55° in this case). Conjugate Riedel shears (R′) are dextral strike-slip faults that form at an angle of 75° to the main line of faulting (a strike of 360° in this case). P shears develop in a later stage and form at an angle of 10° to the main line of faulting. The direction of the greatest principal stress bisects the angle between R and R′ (grey arrows) producing thrust faults with strikes of 115°. In the orthogonal direction, extension (white arrows) can generate normal faults with strikes of 205°. Produced using Global Mapper v15.1.5 (www.bluemarblegeo.com) and Surfer 11 (www.goldensoftware.com).

Moreover, the existence of a major tectonic feature in the Canary region connecting the Trans-Alboran Fault System with the Central Atlantic mid-ocean ridge has been suggested by Mantovani *et al*.^57^ (Fig. 5). The Trans-Alboran Fault System, which is a sinistral strike-slip megastructure that is more than 1,000 km long, runs along the High Atlas and the Middle Atlas in NW Africa, crosses the Alboran Sea in the Mediterranean and ends in the town of Alicante (Spain). Mantovani *et al*.^57^ proposed a new plate configuration for a comprehensive explanation of the kinematics of the Africa-Eurasia plate system that is compatible with all the Atlantic and Mediterranean evidence. This plate configuration involves the existence of the Morocco microplate, whose southern limit would be defined by the prolongation of the Trans-Alboran Fault System through the Canary Archipelago to the Atlantis Fracture Zone (Fig. 5a). In this framework, the submarine fault between Tenerife and Gran Canaria could be interpreted as a non-aligned segment of the Trans-Alboran discontinuity. Once more, an extraordinary agreement of our results with the models obtained using other geophysical data can be noted.

It is worth noting that even if the fault between Tenerife and Gran Canaria has not been active in recent time or in the present-day, our magnetic models indicate that it conditioned the ascent of magmas in the central part of the archipelago during the early stages of growth of the island of Gran Canaria.

Previous estimations of the strike of this fault range from NNE-SSW^54^ to NE-SW and ENE-WSW^53^. Our results agree better with the latter orientation, although it must be stressed that each of the suggested trends are compatible when a strike-slip tectonic framework such as that proposed herein is considered. In fact, in a sinistral strike-slip context such as the Trans-Alboran System, a collection of faults with different orientations can develop and coexist (Fig. 5c), including nearly NE-SW sinistral strike-slip faults (Riedel shears), nearly N-S dextral strike-slip faults (conjugate Riedel shears), and nearly E-W sinistral strike-slip faults (P shears)^58^. In addition, NNE-SSW normal faults and WNW-ESE thrust faults can also be present. The resulting stress regime would be characterized by compression along a nearly NNE-SSW direction and extension along a nearly WNW-ESE direction. This tectonic setting is also supported by first-order models of the present-day plate-scale stress field in the Canary region^59^. Numerical simulations that included the mechanical effect of the Atlantis Fracture Zone through the Canary Islands revealed extensional and strike-slip stresses together with compressional stresses in the vicinity of the central islands, which are also compatible with the focal mechanism of the 1989 earthquake (see Fig. 7b and 7c of Geyer *et al*.^59^).

In accordance with this, it can be noted that the recent modelling of aeromagnetic data for the island of Tenerife revealed an important intrusive body elongated along an E-W direction, suggesting the presence of a major tectonic feature in the crust during the early stages of growth of this island^29^. This E-W tectonic trend was recognized as a major feature in the Canary region by Banda *et al*.^60^ and Marinoni & Pasquarè^18^ and could also be related to the prolongation of the Trans-Alboran discontinuity through the Canary Archipelago. These results clearly point to the presence of an important fault system that is compatible with a strike-slip framework in the central part of the Canary Archipelago and suggest a strong tectonic control on the ascent of magmas, at least in Gran Canaria and Tenerife.

At this point, we want to pay attention to the following statement about the implications of the high eruptive rates observed in Gran Canaria that was written by McDougall & Schmincke^50^: *“The rapid construction of these volcanoes suggests that not only were large volumes of basaltic magma available but that there was a ready path for them to reach the earth’s surface”*. Once more, the presence of crustal fractures seems to be necessary to explain the characteristics of the evolution of this island.

A link between the tectonics and magmatism of the Atlas Mountains in Africa and the origin of the Canary Islands has been proposed in previous studies^4,10,53^. The Atlas Mountains in NW Africa were built through the tectonic inversion of a failed rift system that formed during the opening of the Central Atlantic in the early Mesozoic. The rift was deformed by compression due to African-Eurasian convergence over the last 45 My^61^.

With the information available as of 2000, Anguita & Hernán^10^ remarked on the following analogies between the Canary Islands and the Atlas Mountains: a) the Cenozoic Atlas parental magmas and the Canary magmas share a common origin as deduced from seismic tomography data; b) tectonic features in both regions show remarkable similarities, both with regard to type (mainly strike-slip) and strike (i.e. NE-SW and ENE-WSW); and c) the age of volcanism and the compositional range of volcanic rocks in the Atlas region and in the Canary Archipelago are roughly similar. Based on these correlations, Anguita & Hernán^10^ hypothesized that the fractures inherited from a failed rift arm that developed in NW Africa during the opening of the Atlantic set the path that allowed magma from the mantle plume to reach the surface.

Recent investigations revealed that the uplift of the Atlas Mountains cannot be explained only through inversion tectonics and crustal thickening, but that it also required mantle upwelling^62–65^. The mantle thermal anomaly beneath the Atlas region seems to be connected with the Canary plume through a lithospheric corridor related to the delamination of the mantle lithosphere. This corridor allowed mantle material to travel laterally more than 1500 km from the Canary region to the western Mediterranean over the last 15 My^65,66^. New seismic investigations suggest that this sub-lithospheric channel is a discontinuous structure made up of multiple localized low-velocity zones beneath the Canary Islands, the Atlas Mountains and the Alboran Sea in the Mediterranean^67^.

Citing the works of Uchupi *et al*.^68^ and Froitzheim *et al*.^69^, Anguita & Hernán^10^ identified four uplift periods in the Atlas Mountains and compared them with the ages of magmatic events on the island of Fuerteventura. They observed a temporal alternation of the periods of magmatism in the islands and of compressional stresses in the Atlas Mountains and Atlantic, and they suggested that the fractures would serve as conduits for the magma during the tensional periods and that they would cause uplift of the islands as sets of flower structures during the compressive epochs.

Recent papers about this issue ascribed the uplift of the Atlas region only to two distinct episodes dated to the Middle to Late Eocene–Oligocene and the Late Miocene–Pliocene^64,70^. Therefore, the idea of correlating the occurrence of magmatism only with extensional tectonics in the Atlas region seems weak. In fact, although volcanism is traditionally thought to require an extensional state of stress in the crust, recent data demonstrated that volcanism also occurs in compressional tectonic settings associated with reverse and strike-slip faulting (see Tibaldi *et al*.^71^ and the references therein). This idea was suggested for the Canary Islands by Staudigel *et al*.^72^, who explained that the N-S striking dykes within the La Palma basal complex had formed from a regional N-S compressive stress field related to the collision between the African and Eurasian plates. Therefore, the different episodes of volcanism in the Canary Islands could have occurred in a framework of both extensional and compressional tectonic regimes.

Following Anguita & Hernán^10^, the main obstacle for the acceptance of a genetic relationship between the Canary Islands and the Atlas chain has been the lack of continuous faults connecting both areas. A possible explanation for this is the presence of a thick sedimentary layer (with a thickness of nearly 10 km) between the Canary Archipelago and the African coast, which could be responsible for the seismic gap in this area^73^.

Considering the above discussion, we are certain that all of the available data together with our results of magnetic modelling interpreted in the geodynamical framework proposed by Mantovani *et al*.^57^ strongly support the link between Atlas tectonism and the volcanism in the Canary Islands.

## Conclusions

The 3-D inverse modelling of a linear magnetic anomaly measured over the NW submarine edifice of the volcanic island of Gran Canaria revealed a large, reversely magnetized, elongated structure following an ENE-WSW orientation. It must be stressed that the ambiguities inherent in our magnetic inversion approach (mainly with regard to the parameters defining the source magnetization vector) have no effect on the final results or conclusions of our work since the elongated shape and the orientation of the magnetic source are shared among each of the possible models. In addition, the aeromagnetic data used here have the advantage of being the only geophysical dataset with good resolution that covers both the marine and the subaerial part of NW Gran Canaria. This makes our modelling especially valuable.

We interpret the magnetic source as a sill-like magmatic body emplaced during the submarine stage of growth of this volcanic island with a volume that could represent up to about 20% of the total volume of the island. The elongated shape of this magmatic body suggests the existence of a major crustal fracture in the central part of the Canary Archipelago between Tenerife and Gran Canaria. This fracture would have favoured the rapid ascent and emplacement of magmas during a time interval with duration from 0.5 to 1.9 My, prior to 16 Ma, during which the polarity of the Earth’s magnetic field was reverse. The location and orientation of the presumed fracture agrees with those proposed by Bosshard & MacFarlane^53^ and Mézcua *et al*.^54^ based on seismic and gravity data. The location is also consistent with the southern limit of the Morocco microplate that was defined as the prolongation of the Trans-Alboran fault system from Africa to the Atlantis Fracture Zone through the Canary Archipelago and whose existence was proposed by Mantovani *et al*.^57^ to explain the kinematics of the African and Eurasian plates. Therefore, we propose that the genesis of the Canary Islands was strongly conditioned by a strike-slip tectonic framework probably connected with Atlas tectonism, as suggested by Anguita & Hernán^10^, although volcanism did not necessarily develop during periods of extensional tectonics. These results do not contradict the hotspot theory for the origin of the Canary magmatism, but they undoubtedly introduce the role of regional crustal tectonics on the genesis of these islands to explain where and how those magmas both reached the surface and built the volcanic edifices.

### Data availability

The models generated during the current study are available from the corresponding author on reasonable request.

## Electronic supplementary material

Supplementary figures
