# Supplementary material for: Aeromagnetic anomalies reveal the link between magmatism and tectonics during the early formation of the Canary Islands
Source: Sci Rep. 2018 Jan 8;8:42. doi: 10.1038/s41598-017-18813-w (PMC5758788; doi:10.1038/s41598-017-18813-w)
Supplement: Supplementary file 1 — Supplementary figures [file 41598_2017_18813_MOESM1_ESM.pdf]

Supplementary information

Aeromagnetic anomalies reveal the link between magmatism and tectonics during the early formation of the Canary Islands

Isabel Blanco-Montenegro* [1,4], Fuensanta G. Montesinos[2,4] and José Arnoso[3,4]

1 - Universidad de Burgos, Departamento de Física, Escuela Politécnica Superior, Avda. de Cantabria s/n, 09006 Burgos, Spain; e-mail: iblanco@ubu.es

2 - Facultad de Matemáticas, Universidad Complutense de Madrid, Plaza de Ciencias 3, 28040 Madrid, Spain; e-mail: fuensanta.gonzalez@mat.ucm.es

3 - Instituto de Geociencias (CSIC, UCM), Facultad de Medicina (edificio Entrepabellones 7 y 8), c/ Doctor Severo Ochoa 7, 28040 Madrid, Spain; e-mail: arnoso@ucm.es

4 - Research Group 'Geodesia', Facultad de Matemáticas, Universidad Complutense de Madrid, Plaza de Ciencias 3, 28040 Madrid (http://www.mat.ucm.es/grupogeodesia/)

* Corresponding author

Supplementary figures

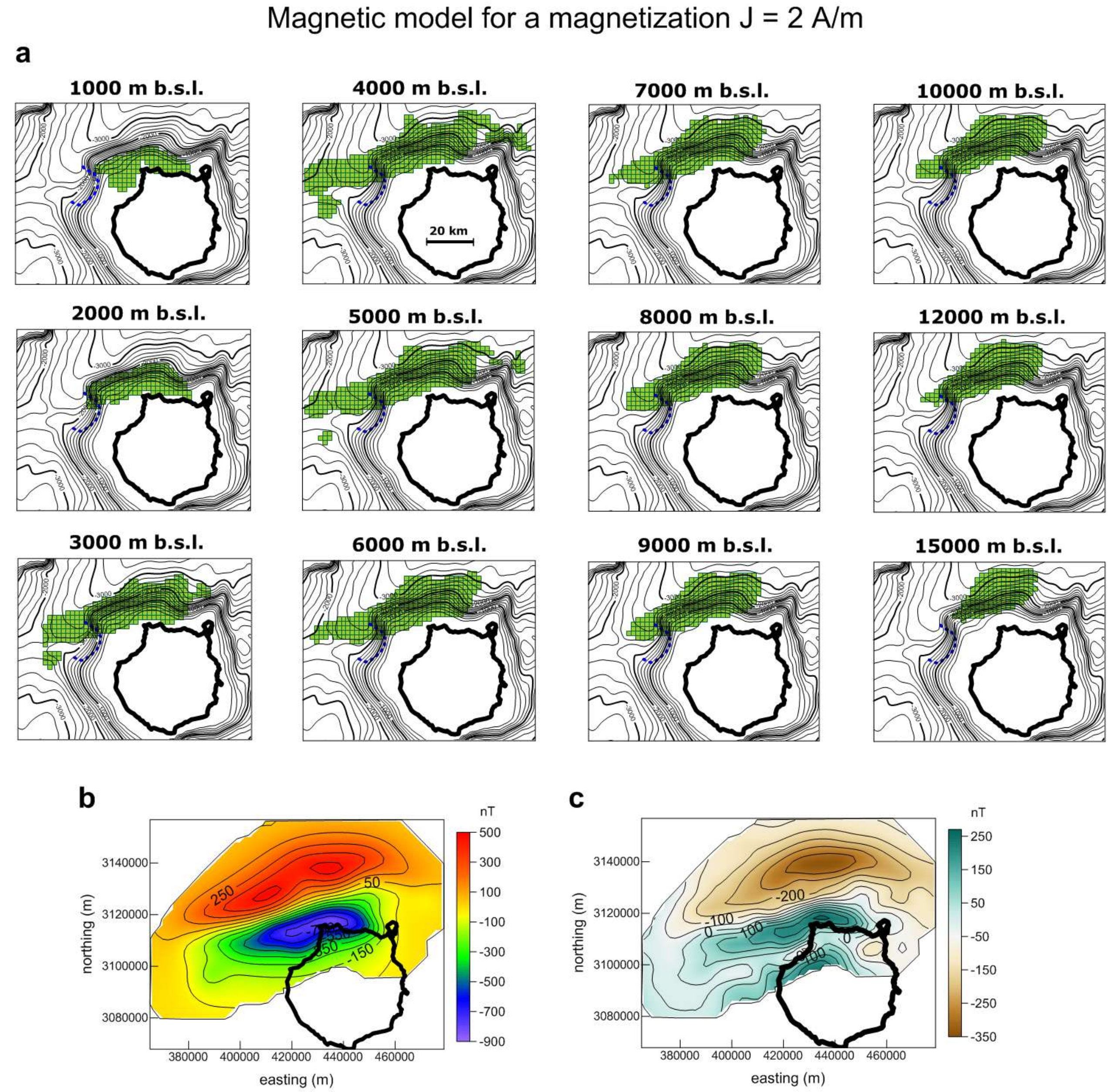

Figure S1. (a) Horizontal sections of the magnetic source obtained through the inversion of the aeromagnetic data for a magnetization **J = 2 A/m**. The thick black line shows the coastline of the island. The dashed blue line marks the position of the submarine landslide scar identified by Krastel & Schmincke[47]. (b) Magnetic anomaly from the inversion model shown in a. (c) Residual magnetic anomaly obtained as the difference between the observed magnetic anomaly shown in Fig. 2b and the magnetic anomaly of the inversion model shown in panel b. Coordinates correspond to the UTM projection (zone 28N).

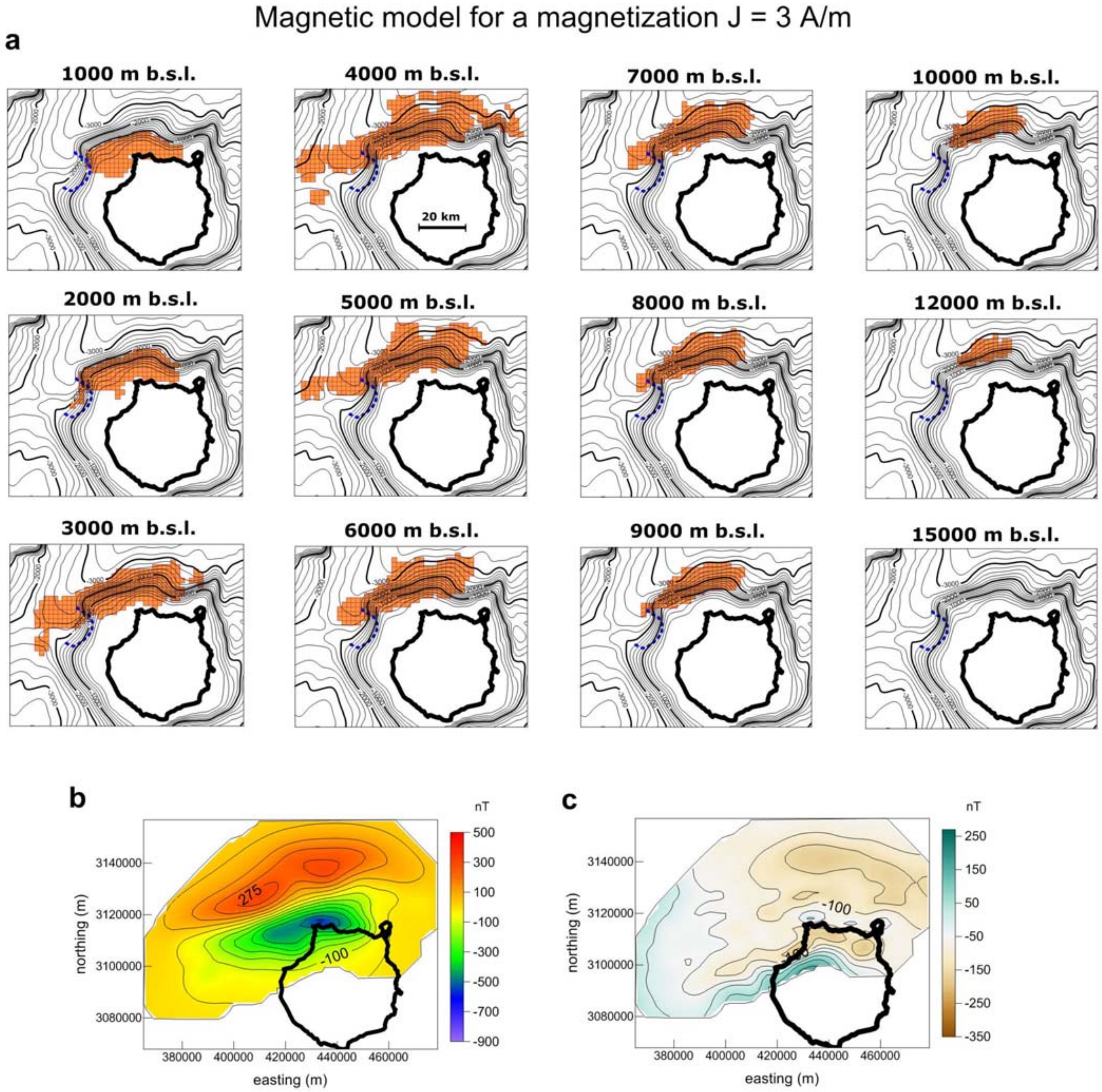

Figure S2. (a) Horizontal sections of the magnetic source obtained through the inversion of the aeromagnetic data for a magnetization **J = 3 A/m**. The thick black line shows the coastline of the island. The dashed blue line marks the position of the submarine landslide scar identified by Krastel & Schmincke[47]. (b) Magnetic anomaly from the inversion model shown in a. (c) Residual magnetic anomaly obtained as the difference between the observed magnetic anomaly shown in Fig. 2b and the magnetic anomaly of the inversion model shown in panel b. Coordinates correspond to the UTM projection (zone 28N).

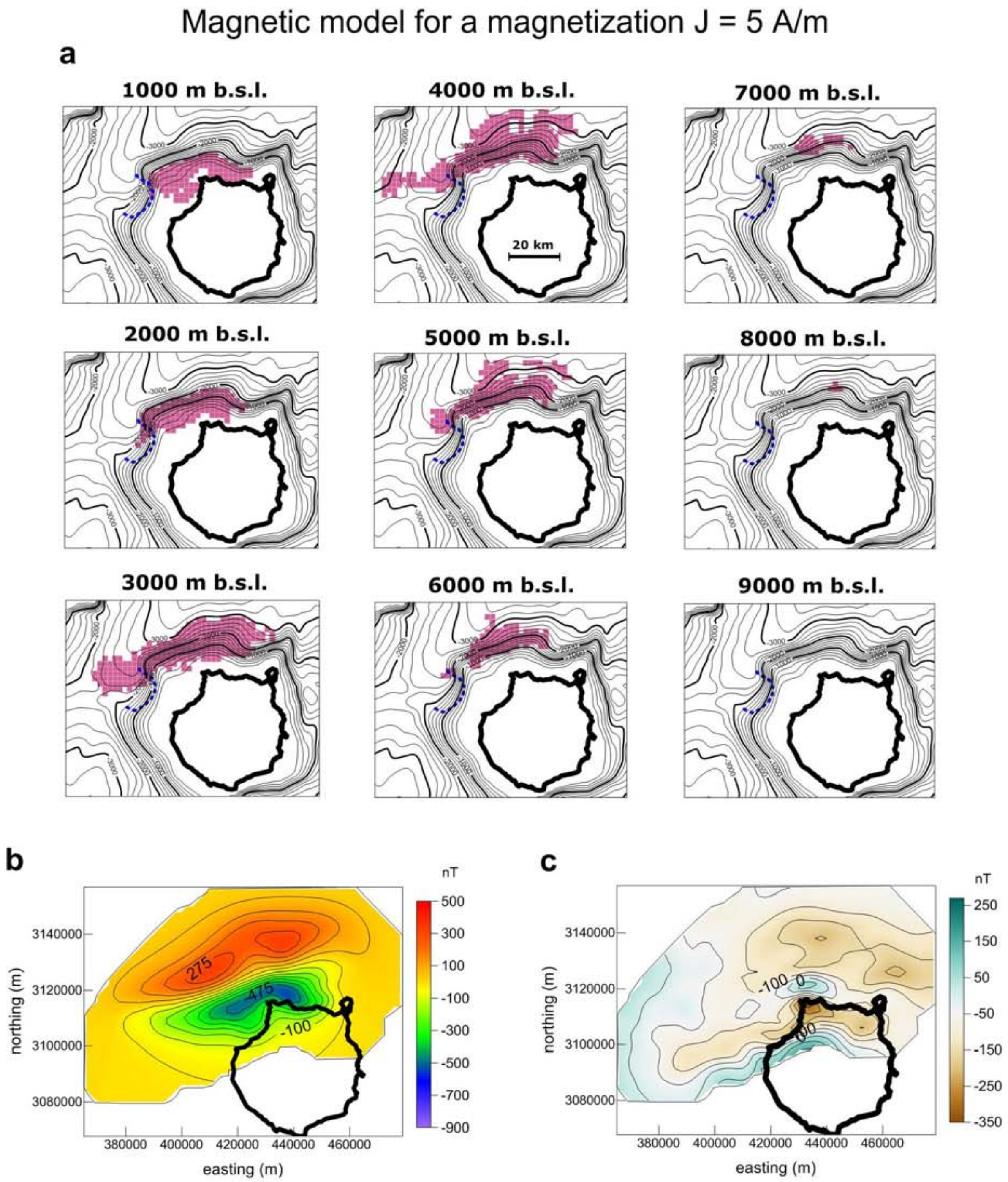

Figure S3. (a) Horizontal sections of the magnetic source obtained through the inversion of the aeromagnetic data for a magnetization **J = 5 A/m**. The thick black line shows the coastline of the island. The dashed blue line marks the position of the submarine landslide scar identified by Krastel & Schmincke[47]. (b) Magnetic anomaly from the inversion model shown in a. (c) Residual magnetic anomaly obtained as the difference between the observed magnetic anomaly shown in Fig. 2b and the magnetic anomaly of the inversion model shown in panel b. Coordinates correspond to the UTM projection (zone 28N).

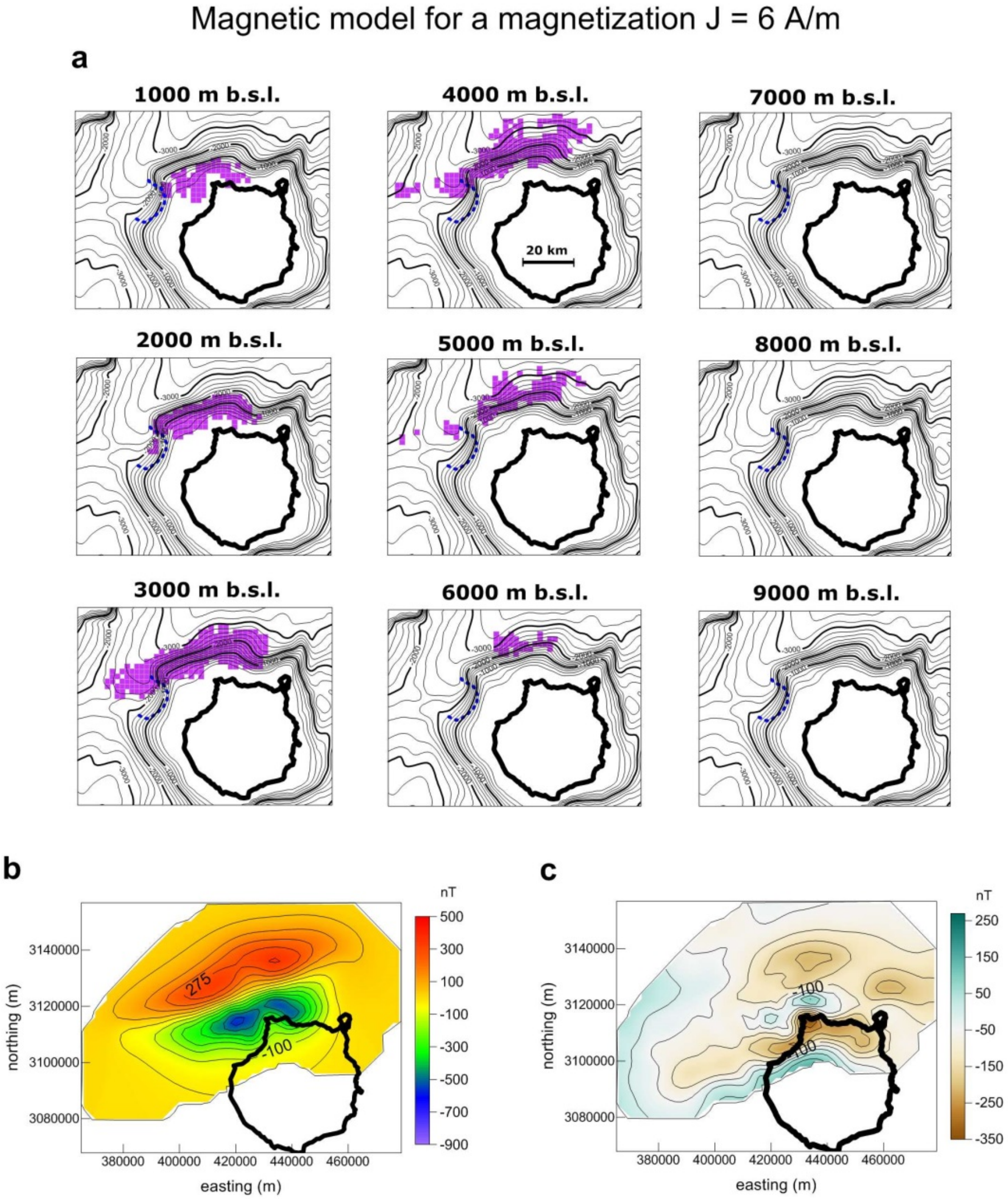

Figure S4. (a) Horizontal sections of the magnetic source obtained through the inversion of the aeromagnetic data for a magnetization **J = 6 A/m**. The thick black line shows the coastline of the island. The dashed blue line marks the position of the submarine landslide scar identified by Krastel & Schmincke[47]. (b) Magnetic anomaly from the inversion model shown in a. (c) Residual magnetic anomaly obtained as the difference between the observed magnetic anomaly shown in Fig. 2b and the magnetic anomaly of the inversion model shown in panel b. Coordinates correspond to the UTM projection (zone 28N).

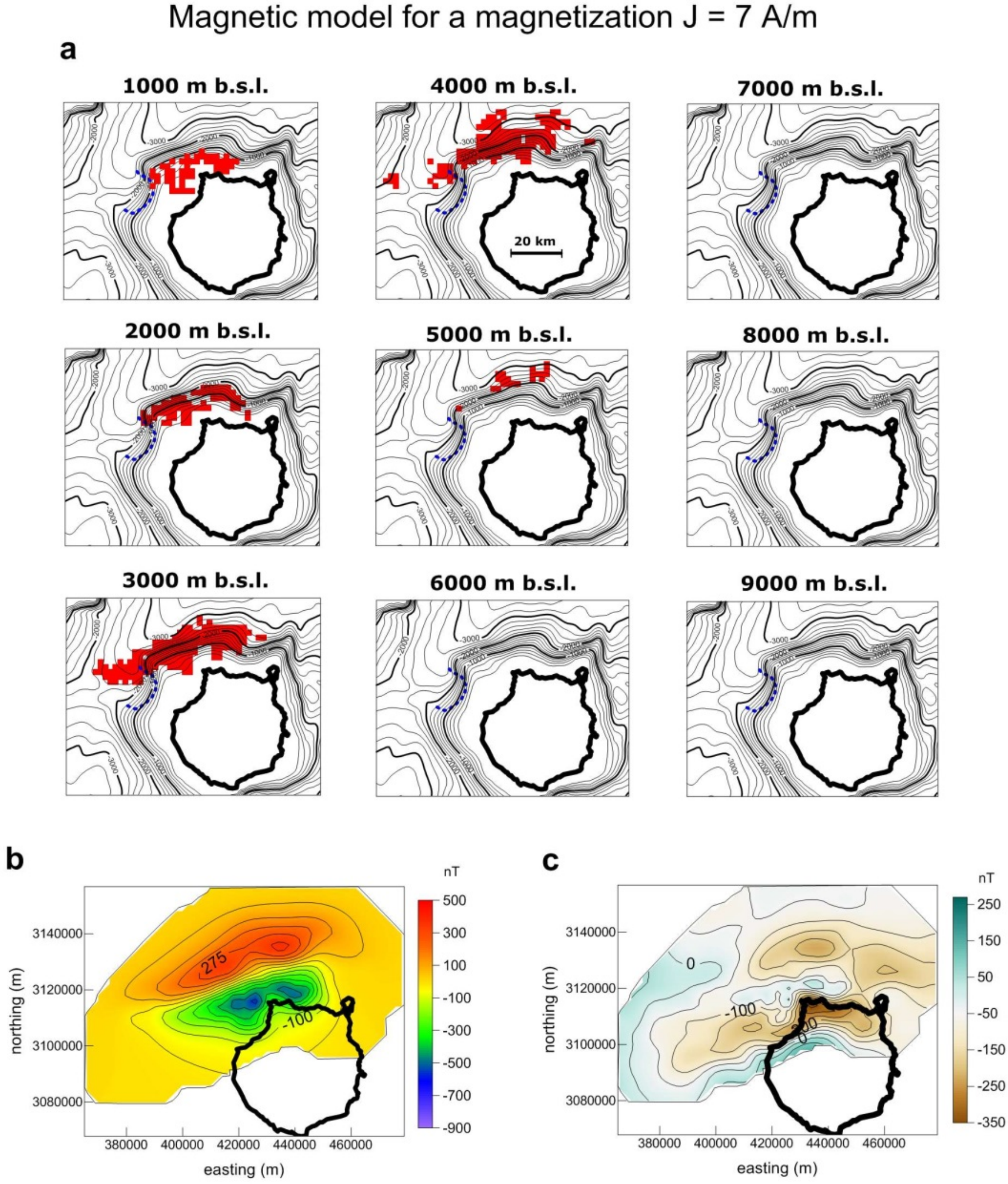

Figure S5. (a) Horizontal sections of the magnetic source obtained through the inversion of the aeromagnetic data for a magnetization **J = 7 A/m**. The thick black line shows the coastline of the island. The dashed blue line marks the position of the submarine landslide scar identified by Krastel & Schmincke[47]. (b) Magnetic anomaly from the inversion model shown in a. (c) Residual magnetic anomaly obtained as the difference between the observed magnetic anomaly shown in Fig. 2b and the magnetic anomaly of the inversion model shown in panel b. Coordinates correspond to the UTM projection (zone 28N).

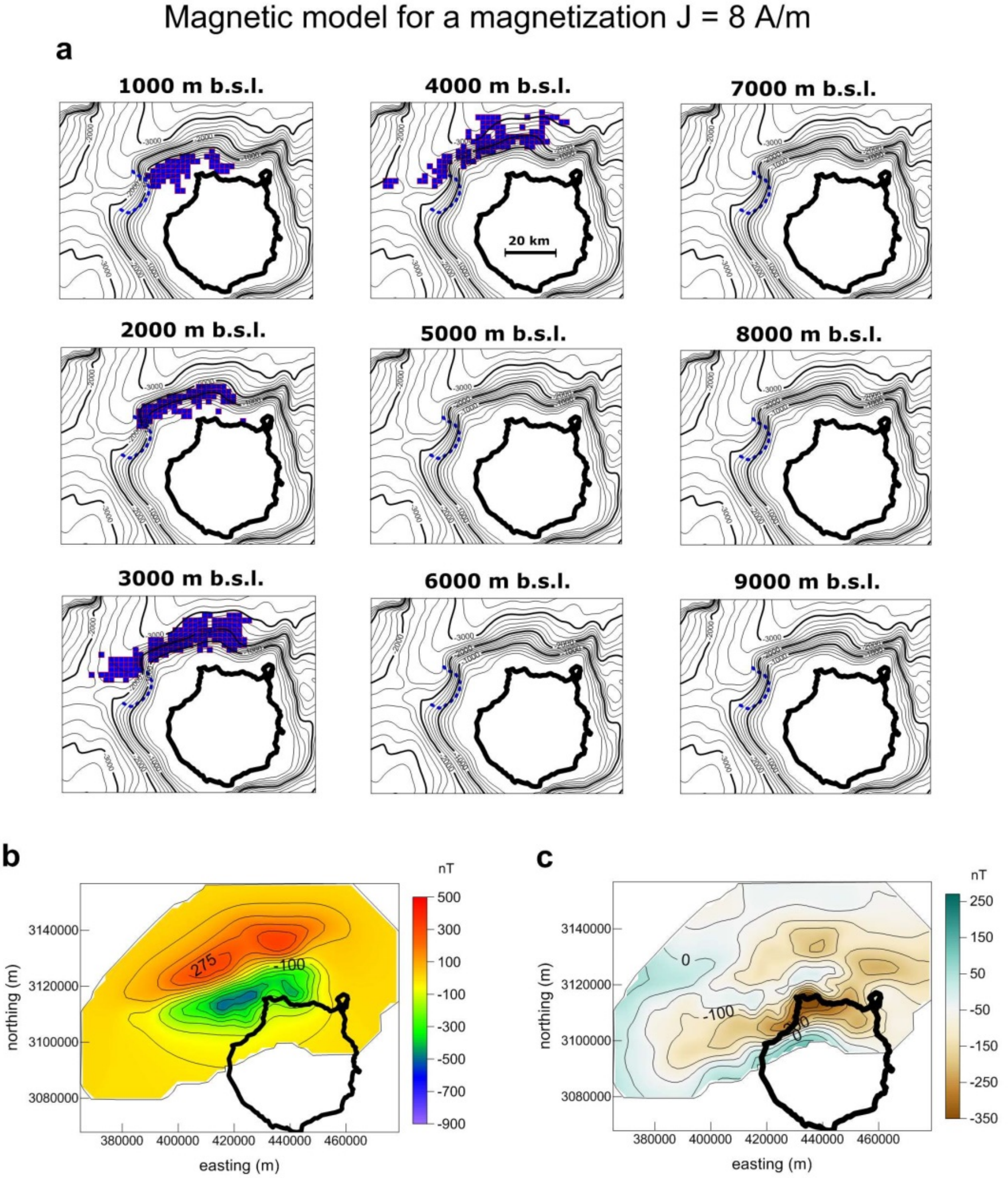

Figure S6. (a) Horizontal sections of the magnetic source obtained through the inversion of the aeromagnetic data for a magnetization **J = 8 A/m**. The thick black line shows the coastline of the island. The dashed blue line marks the position of the submarine landslide scar identified by Krastel & Schmincke[47]. (b) Magnetic anomaly from the inversion model shown in a. (c) Residual magnetic anomaly obtained as the difference between the observed magnetic anomaly shown in Fig. 2b and the magnetic anomaly of the inversion model shown in panel b. Coordinates correspond to the UTM projection (zone 28N).

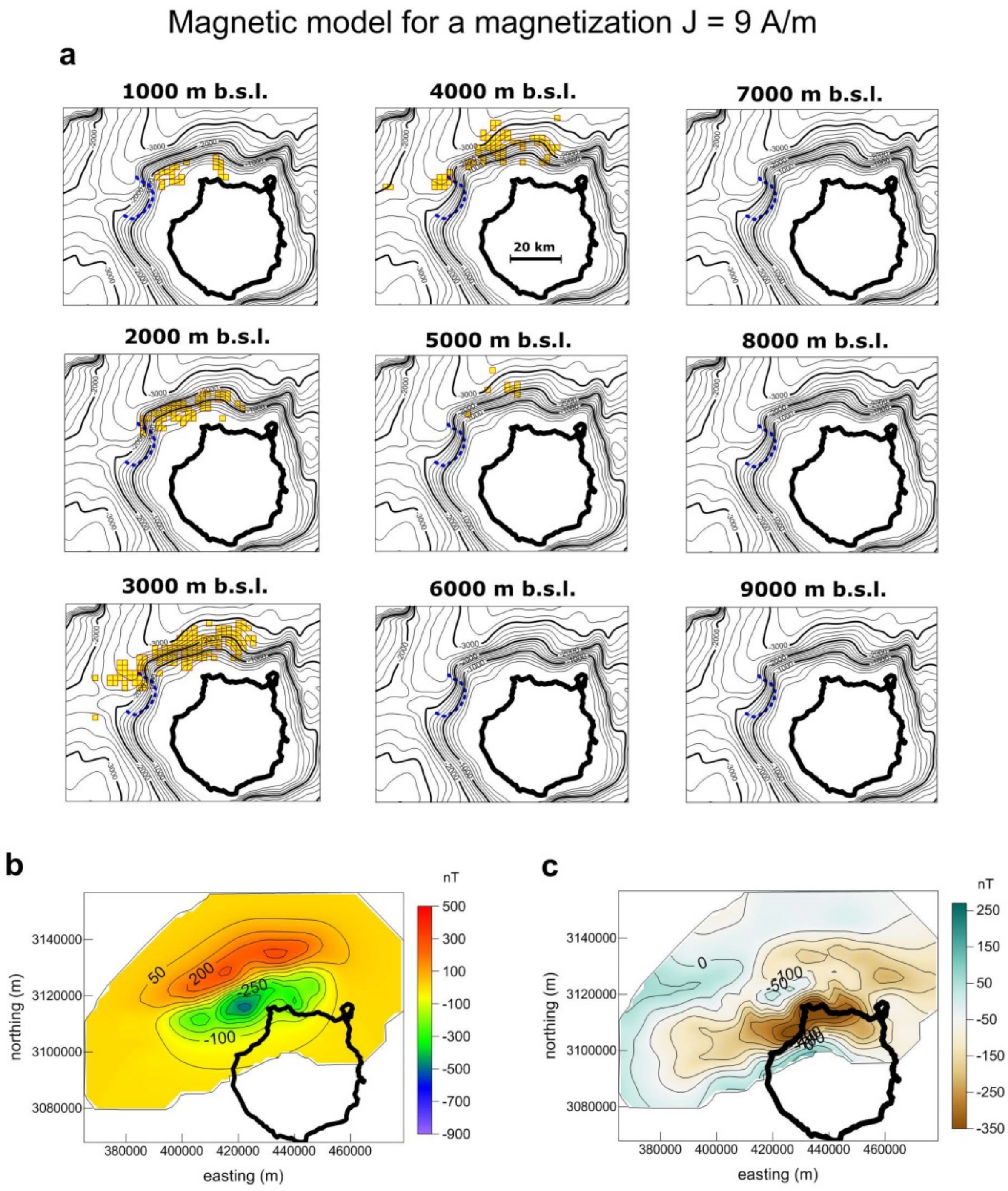

Figure S7. (a) Horizontal sections of the magnetic source obtained through the inversion of the aeromagnetic data for a magnetization **J = 9 A/m.** The thick black line shows the coastline of the island. The dashed blue line marks the position of the submarine landslide scar identified by Krastel & Schmincke[47]. (b) Magnetic anomaly from the inversion model shown in a. (c) Residual magnetic anomaly obtained as the difference between the observed magnetic anomaly shown in Fig. 2b and the magnetic anomaly of the inversion model shown in panel b. Coordinates correspond to the UTM projection (zone 28N).

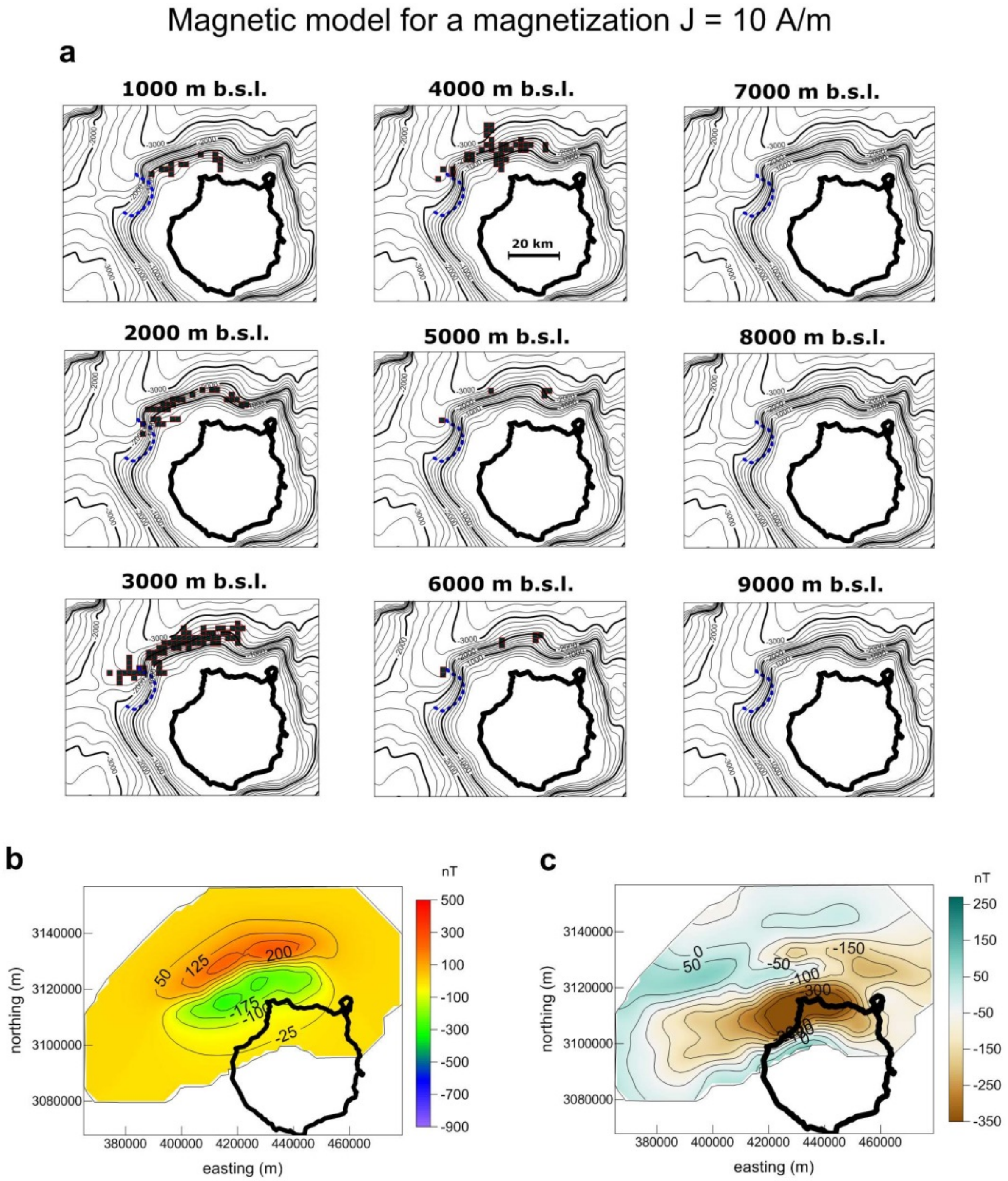

Figure S8. (a) Horizontal sections of the magnetic source obtained through the inversion of the aeromagnetic data for a magnetization **J = 10 A/m**. The thick black line shows the coastline of the island. The dashed blue line marks the position of the submarine landslide scar identified by Krastel & Schmincke[47]. (b) Magnetic anomaly from the inversion model shown in a. (c) Residual magnetic anomaly obtained as the difference between the observed magnetic anomaly shown in Fig. 2b and the magnetic anomaly of the inversion model shown in panel b. Coordinates correspond to the UTM projection (zone 28N).
